# Support for people who use Anabolic Androgenic Steroids: A Systematic Scoping Review into what they want and what they access

**DOI:** 10.1186/s12889-019-7288-x

**Published:** 2019-07-31

**Authors:** Orlanda Harvey, Steve Keen, Margarete Parrish, Edwin van Teijlingen

**Affiliations:** 1 Bournemouth University, Lansdowne Campus, Royal London House, 109 Christchurch Road, Bournemouth, BH1 3LT UK; 2 Bournemouth University, CMMPH, Bournemouth House, 19 Christchurch Road, Bournemouth, BH1 3LH UK

**Keywords:** Androgenic anabolic steroids (AAS), Image and performance enhancing drugs (IPED), Support, Harm minimisation, Advice, Information, Needle and syringe Programmes (NSPs)

## Abstract

**Background:**

Since there is a paucity of research on support for people using Anabolic Androgenic Steroids (AAS), we aimed to identify and synthesise the available evidence in this field. Gaining an understanding of the support both accessed and wanted by recreational AAS users will be of use to professionals who provide services to intravenous substance users and also to those working in the fields of public health and social care, with the aim to increase engagement of those using AAS.

**Methods:**

A systematic scoping review of the literature to explore and identify the nature and scope of information and support both accessed and wanted by non-prescribed AAS users. Any support services or information designed to help people who use AAS were considered.

**Results:**

We identified 23 papers and one report for review, which indicated that AAS users access a range of sources of information on: how to inject, substance effectiveness, dosages and side effects, suggesting this is the type of information users want. AAS users sought support from a range of sources including medical professionals, needle and syringe programmes, friends, dealers, and via the internet, suggesting that, different sources were used dependent on the information or support sought.

**Discussion:**

AAS users tended to prefer peer advice and support over that of professionals, and access information online via specialist forums, reflecting the stigma that is experienced by AAS users. These tendencies can act as barriers to accessing services provided by professionals.

**Conclusions:**

Support needs to be specific and targeted towards AAS users. Sensitivity to their perceptions of their drug-use and the associated stigma of being classified in the same sub-set as other illicit drug users is relevant to facilitating successful engagement.

**Electronic supplementary material:**

The online version of this article (10.1186/s12889-019-7288-x) contains supplementary material, which is available to authorized users.

## Background

In the UK, just under 54,000 16–59 year-olds reported having used Anabolic Androgenic Steroids (AAS) in 2015/2016 [1]. Although representing only a small minority of all substance users, this is probably underreported due to the illegality of supply and the use of self-reported data. A simultaneous increase in the use of needle and syringe programmes (NSPs) by people using Image and Performance Enhancing Drugs (IPED) (including AAS) has also been noted [2]. NSPs provide harm minimisation services to people who inject substances, which includes handing out injecting paraphernalia, offering advice on safe injecting and harm minimisation and sometimes screening for Blood Borne Viruses (BBVs) [3]. Since the late 1980s NSP support has become an established service for AAS users [4], in one study of 500 users [5] 99.2% reported using injectable AAS or a combination of injectable and oral substances and a recent UK survey of 684 AAS users, 85% of users injected IPED, and steroids were the most commonly used IPED [6].

AAS use is linked with negative physical health effects, such as testicular atrophy, liver toxicity, dermal scarring, cognitive problems, gynaecomastia, muscle damage, myocardial injuries, infertility [7], and BBVs [8]. AAS users are at greater risk than non-users of psychological risks such as: mania, delusions, aggressive behaviours, depression, suicide and anxiety [9–14]. Pilot studies have shown that lifetime AAS use may impact on some cognitive processes and the structural features of the brain [15–17].

Further risks include harm from using AAS in combination with illicit substances [18], self-medication [19] and becoming AAS dependent [20]. Importantly, not all AAS users will experience these. Reasons for starting use vary, the most prominent being to gain muscle/strength [21, 22] and historically this has been associated with sport. However, recently a wider range of motivations has been identified including improved appearance, aggression, personal security, psychological well-being (including boosting self-esteem or confidence) or satisfaction, sexual attraction, overcoming depression, curiosity, influence of family, peers and media [23]. People who use substances are the experts in their own use [24], therefore, given the wide range of risks, a variety of motivations (many not mutually exclusive) and the potential for people to become dependent it is important to understand what support people who use AAS wish to receive. Getting their perspectives on ideal support may lead to more effective engagement with services. Additionally, people working with substance users need knowledge of the types of support available, to make changes relevant to their needs and to reduce the risk of harm to self and others [25].

Consequently, this systematic scoping review explores the nature and scope of the information and support accessed and wanted, by investigating two questions:

- What support and information do people using non-prescriptive AAS recreationally access?
- What support and information do these recreational AAS users say they want?

## Methods

Scoping reviews can be helpful in providing one source of information for professionals to develop Practice Guidance [26]. A scoping review follows a systematic process but allows for flexibility, incorporating changes as part of the iterative process [27], and allows for the inclusion of grey literature. To ensure the process was transparent, robust and replicable, the authors followed the Preferred Reporting Items for Systematic Reviews and Meta-Analyses (PRISMA) guidelines [28]. Our protocol is registered in PROSPERO [29].

### Search strategy

The wide variation of terms to describe AAS means that searching the literature is fraught with difficulty and could lead to key studies being left out, as the term ‘IPED’ is often used when covering a wider variety of substances than just steroids such as Human Growth Hormone [30]. Variations on the acronyms included: PIED, PES, PED, APED, NMASS (non-medical Anabolic Androgenic Steroid), and terms such as ‘doping’, ‘testosterone boosters’, ‘prohormones’, ‘ergogenic aids’ ‘designer steroids’ and brand names. The first author tested key words and word groupings, drawn from recent UK Public Health literature.

In June 2018 a search was carried out in EBSCO (Table 1), searching 141 databases. Papers were found in 52 databases (see Additional file 1). Some databases proved irrelevant, but it was useful to take a multi-disciplinary approach as it was difficult to predict where the most pertinent studies might come up. Separate searches on SCOPUS, Google Scholar and reference lists of included articles were also undertaken, as electronic databases may not throw up all available literature [31].

**Table 1:** Systematic review search strategy - search terms

	Search algorithms
	anabolic androgenic OR designer N3 steroid* OR recreat* steroid* OR anabolic steroid* OR anabolic drug* OR Synthe* testosterone OR “Synthe* testosterone” OR “non prescript*” steroid* OR non-prescript* steroid* OR “non-medic*” steroid* OR “non prescri*” N2 steroid* OR non-prescri* N2 steroid* OR “non medic*” N2 steroid* OR non-medic* N2 steroid* OR performance N3 enhanc* drug* or image N3 enhanc* drug* or appearance N3 enhanc* drug* or muscle N3 enhanc* drug* OR muscle N3 develop* drug* or performance N3 develop*drug* OR doping N3 steroid*
NOT	animal* OR mice OR rats OR “guinea pig*” OR spectrometry OR bovine
AND	Support or advice or help or aid or barrier* or information or guidance or intervention* or “needle exchange* or program*”

Inclusion and exclusion criteria (Table 2) were applied initially through a title, abstract and full paper screening. Publications were limited to those in English (due to lack of resources for translation), without geographical restrictions. Irrespective of the study design, articles that met inclusion criteria were reviewed, i.e. populations such as recreational users and non-competitive AAS-using bodybuilders were eligible; there were no age or gender restrictions. The first author screened and reviewed all articles. To validate the search strategy the second author reviewed 10% of articles screened out by title and 20% screened out by abstract. The second, third and fourth authors checked 10% each of articles in the full review.

**Table 2:** Inclusion and exclusion criteria

Inclusion criteria	Exclusion criteria
Studies including populations such as recreational AAS users, non-competitive AAS-using bodybuilders and weightlifters and, AAS users accessing drug services.	Studies involving participants who compete professionally and any study that focuses on competitive sports/athletes or high school athletes
Peer-Reviewed Papers^	Studies on wider drugs prevention interventions or strategies
Qualitative and Quantitative data	Studies that made passing references to participants seeking information but did not clarify the type of support or information including studies which showed an increase in people using NSPs but did not share exactly what they were using them for
Studies where participants were asked about where they access support, advice and information to help them manage their substance use and that identified the types of support and information they were seeking.	Specific medical interventions i.e. efficacy of treatments for side effects
Studies that included data collected on any support (information, advice, service or intervention) designed to support people who use IPED	Studies that focussed on prevention of AAS use and efficacy of such interventions
	Studies that referenced participants attitudes to who they trusted around information but did not specifically state the types of information or support
	Studies that were solely based on recommendations of professionals as to what support and information was needed but where the voice of the AAS user was absent
	Articles not in English
	Studies before the predominance of the internet as a source of information i.e. pre 2001. In 2001 the number of internet users went over 500 million worldwide [32]

*^ due to the limited number of articles found to answer the second question, inclusion criteria were modified to include relevant grey literature from references*

### Literature search

**Fig. 1:**
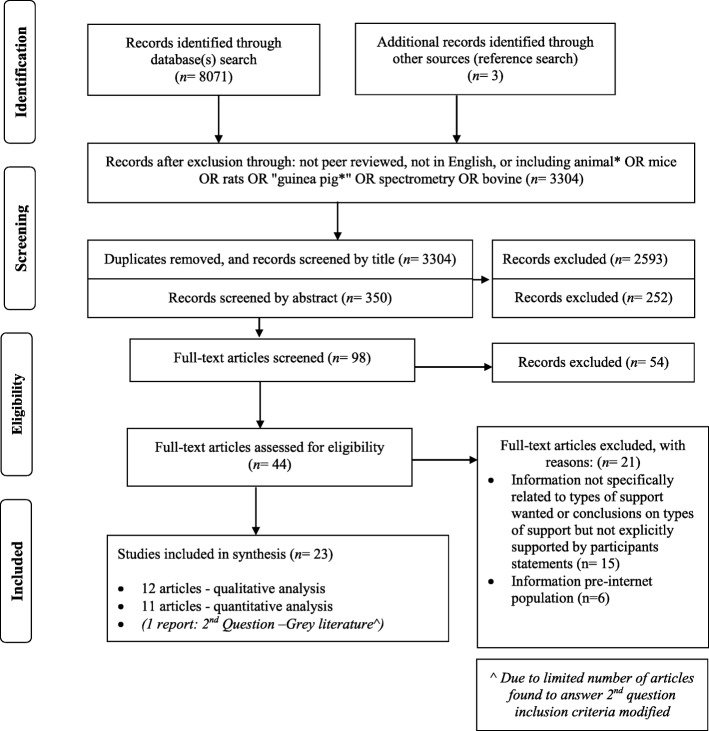
PRISMA 2009 Flow Diagram [34]

Our search found several papers relating to question 1, but few relating to question 2, therefore the search strategy was revised for question 2. Scoping reviews do not necessarily have to rate the quality of the papers [27], however the authors concluded that due to the complexity of identifying participants, such a quality review was of value. Therefore, for question 1, only peer-reviewed documents were included to ensure a level of quality, and this proved fruitful when considering support accessed. However, for question 2, only nine papers gave limited information on support wanted, therefore the authors searched the references of the included articles for grey literature (non-peer reviewed) that might include qualitative data on ‘ideal support’. One report that specifically sought information relating to ideal support wanted was identified [33]. Acknowledging this report was not peer reviewed, the authors felt the information contained was of value and relevant to the second question. Figure 1 outlines the search strategy.

### Data extraction and analysis

Information regarding support and advice, population, substance use, study aims, recruitment methods, methodology and demographics was extracted by the first author and 30% of data extraction forms were crossed checked by co-authors. Reviewer agreement on inclusion and exclusion criteria was 100%. Both qualitative and quantitative data were included. Due to the different types of articles, three quality assessment tools were used: The CASP Checklist [35] for qualitative articles (Table 3).

**Table 3:** Summary of papers included: Qualitative studies

First author, year & reference	Country	Participants defined, (age range/mean), gender	No. of participants	Type of data #	Sources potential bias & limitations	Quality review~
Maycock (2005) [36]	Australia	Used or had used AAS and dealers, men	42 AAS users, 22 dealers	#Qualitative: Participant observation (147), interviews include longitudinal (10 over 3 years)	Purposive sampling	**
Grogan (2006) [37]	UK	Use(d) AAS, 5 men, 6 women	11	#Qualitative: interviews	Small sample	***
Skårberg (2008) [38]	Sweden	Addiction clinic patients who use(d) AAS, 4 men, 2 women	6	Qualitative interviews: case-study	Sought help for AAS use. Small sample	**
Kimergård (2014) [39]	England & Wales	AAS users and harm reduction service providers (mean = 34), men	24	Qualitative: semi-structured interviews	Bias towards those showing positive health behaviours	***
Kimergård (2014) [3]	England & Wales	Used or had used AAS, men	24	#Qualitative: semi-structured interviews	same study as above	***
Kimergård (2015) [22]	England & Wales	AAS users, men	24	#Qualitative: semi-structured interviews	same study as above	***
Van Hout (2015) [40]	UK	IPED users, men	20	#Qualitative: in-depth interviews	Privileged access recruitment^	***
Dunn (2016) [41]	Australia	Used or had used AAS, 19 men and 2 women	21	#Qualitative: semi-structured interviews	Voucher for taking part; one region (non-rural), length of interviews varied	***
Griffiths (2016) [42]	Australia	Used or had used AAS, 24 men 2 women	26	#Qualitative: semi-structured interviews	– same study as above	***
Hanley Santos (2017) [43]	UK	AAS users, 21 men 1 woman	22	Qualitative: semi-structured interviews	Bias towards those showing positive health behaviours - £10 given	***
Tighe (2017) [44]	Australia	Specialist forum users, (none), unknown	450 unique avatars	Qualitative: threads from 3 Online forums: 134 threads: 1716 posts	Australian sites yet people from other countries on forums	***
Greenway (2018) [45]	UK	AAS Users, male	8	Qualitative: interviews	Sample bias, one NSP	***

~ Quality Review: Qualitative studies: CASP Checklist for Qualitative Research [34] was used: *** 90% boxes checked as yes, evident, ** if equal to or greater than 70% checked. ^The authors acknowledged that one interviewer had privileged access, here that is likely to be an insider within this sub-community [49] #where practicable data analysed from studies which included dealers or professional services providers, only findings from AAS users have been included

For the quantitative studies the Quantitative Review Methodology tool by Davids and Roman [46] was adapted. To assess the quality of the grey literature, the quantitative and qualitative elements were individually assessed using the aforementioned assessment tools, then the Mixed Methods Appraisal Tool [47] was used, to assign an overall quality score. Studies scoring ** or above (*** 67–100% & **34–66% score) were included (Table 4) and no studies were excluded on the basis of quality.

**Table 4:** Summary of papers included: Quantitative and mixed methods studies

First author, year & reference	Country	Participants defined, (age range/mean), gender	No. of participants	Type of data #	Sources, potential bias & limitations	Quality review~
Parkinson (2006) [5]	USA	AAS users, 494 men 6 women	500	Quantitative: web-based questionnaire	Web-based, self-selected, self-report	**
Cohen (2007) [48]	US	AAS users (Non-medical), men	1955	Quantitative: web-based survey	Online population	***
Larance (2008) [49]	Australia	IPED users, men	60	#Quantitative: cross -sectional structured Interviews	Self-selecting sample, purposive recruitment strategies, self-reports	***
Al-Falasi (2009) [50]	UAE	AAS users (34 male) and non-AAS users (129 male & female), age range not specific	154	Quantitative: Self-administered questionnaire	Self-report, small sample size, selective bias	**
Bojsen-Møller (2010) [51]	Denmark	General public (incl AAS users), (not given for AAS queries subset), 284 men, 40 women	374	Quantitative: Anti-Doping Hotline Enquires: web and phone queries (subset AAS use)	Self-selected, missing data for AAS users’ subset	**
Hope (2013) [52]	England & Wales (UK)	Injectors of IPED (NSPs), (*n* = 347 mean = 28 [not all gave age]), men	395	Quantitative: unlinked-anonymous cross-sectional biobehavioural survey (oral fluid sample)	NSPs as settings	***
Hope (2013) [19]	England & Wales (UK)	Injectors of IPED (NSPs), (*n* = 319, mean: 28 [not all gave age]), men	366	Quantitative: unlinked-anonymous cross-sectional biobehavioural survey (oral fluid sample)	same study as above	***
van Beek (2015) [53]	Australia	Injectors of IPED (NSPs), (mean = 32.6), men	103	Quantitative: Self-administered survey	Recruited from 2 public healthcare providers	***
Jacka (2017) [54]	Australia	Injectors of IPED, (median 27), men	100000 occasions	Queensland NSP Minimum dataset	NSPs as settings	***
Rowe (2017) [8]	Australia	Injectors of IPED, (mean = 28.8), men	605	Quantitative: Self-administered questionnaire	NSPs as settings	***
Zahnow (2017) [55]	Global	AAS users, 253 men & 59 women (no exact No. after exclusion criteria applied)	195 AAS users with adverse effects	Quantitative: Sub-section of global drug survey – online	Self-nominating, online only	**
*Dennington (2008)* [34]	*Australia*	*IPED users, 61 men, 1 woman, 7 trans, 24, key informants*	*69 (+ 24)*	*#Mixed Methods: semi-structured interviews collecting quantitative and qualitative data*	*Report: not peer reviewed. Data sets not integrated*	**

~ Quality Review: Davids and Roman’s [47] Quantitative Review Methodology. Appraisal Score: *** 67–100% & **34–66% score. #where practicable data analysed from studies which included dealers or professional services providers, only findings from AAS users have been included

There have been several challenges when identifying and reviewing the literature. The number of different terms that cover AAS is inconsistent (Table 1). Identifying purely recreational users was difficult due to a lack of granularity when studies consider AAS/IPED use e.g. terms such as bodybuilder, weightlifter and athlete were utilised both for competitive and recreational use. Not all studies identified whether participants used solely AAS or in combination with other IPED. Due to the heterogeneous nature of the data this review takes a narrative approach. Moreover, unless clearly stated as AAS use within the study, the generic term IPED will be used.

Analysis was mixed method as scoping reviews can incorporate numerical summaries alongside thematic analysis of qualitative data [56]. Initially tabulations were used for the quantitative data, which led to the identification of specific categories such as BBV checks and acquisition of injecting equipment. Thematic analysis was conducted in an inductive way, each article was read to identify types of information and support and then categorised into type 1 (information or support accessed) or type 2 (information or support wanted). The research team met frequently to discuss the emerging themes, which led to the identification of three overarching themes: harm minimisation, research and information and support for health concerns. Then sub categories were identified based on the type of information or support. It was challenging to identify the type of information participants were searching for and in these instances the authors coded this data as ‘*seeking of general information on IPED use’.*

## Results

For question 1, twenty-three papers: eleven quantitative articles (nine studies) and twelve qualitative articles (nine studies) were included as for several papers the same data set was used to explore different questions related to the use of AAS (Fig. 1). For question 2, nine studies were included and one report.

Sample sizes for IPED-using participants ranged from six to 1955. All studies incorporated data on information or support accessed and the majority were self-reported. Ten studies featured only male AAS-using participants. In the seven studies where gender was recorded there were only twenty women, and one study of 253 men, and 59 women, did not report the gender split after participants who reported no adverse effects were excluded, leaving a mixed-gender sample of 195. One study included women but only as non AAS-users [49]. Two studies: one on an anti-doping hotline [50] and another on online forum posts [44] had incomplete demographic data and one did not record discreet visits of NSP services [51].

### Information and support sought

IPED users sought different types of information and support from a range of potentially overlapping sources: NSPs, pharmacies, doctors, sexual health clinics, other medical professionals, peers, coaches/trainers, friends, dealers, family, the internet, specialist online fora, experienced users, steroid guides in gyms, underground books, online videos and addiction clinics (Table 5).

**Table 5:** Data by type of information or support - Harm minimisation

Type of information / support	Support sought from (if given)	Article reference
Acquisition of injecting equipment	Dealer(s)	[36, 40, 43]
	NSPs	[19, 22, 39–41, 43, 51–54]
	Chemist/Pharmacy	[40, 41, 43, 52, 54]
	Doctor(s)	[39, 52, 54]
	Friends(s)/Peer(s)/Social Network	[41, 43, 52, 54]
	Steroid Clinic(s)	[22, 39]
	Gym/Outreach services in Gyms	[39, 54]
	Online/Websites	[40, 54]
	Anti-Aging clinic(s)	[41]
	Outreach service/Other	[22, 52]
Guidance on how to inject and safer injection practices	Dealer(s)/Supplier(s)	[8, 36, 40, 43]
	Friend(s)/Peer(s)/Experienced Gym mate(s)/Other AAS user(s)/Family	[8, 38–40, 46, 53]
	Self-taught	[52]
	NSPs	[51]
	Online/Websites	[8, 43]
	Leaflets/Other sources	[8, 43]
	Personal trainer(s)	[8]
	Doctor(s)/Nurse(s)	[8, 52, 55]
Blood Borne Virus screening**~**	Hep B and Hep C	[8, 55]
	Hep B (20%), Hep C (18%)	[19]
	Hep C (64%)	[55]
	Hep B (23%), Hep C (22%)	[53]
HiV testing**~**	HiV	[8, 55]
	HiV (31%)	[53]
	HiV (64%)	[54]
	HiV (28%)	[19]

Any data given about access of services that is not linked to AAS/IPED use has not been included in this table. ~Percentage of participants where given

### Harm minimisation and advice

Ten studies evidenced IPED users obtaining injecting equipment from NSPs. However, five studies recruited from harm reduction services [19, 43, 51, 53, 54] and one had predominantly NSP clients [3, 22, 42]. This could explain the prevalence of NSPs as places to access injecting equipment. Hanley Santos and Coomber [43] noted that some reported no difficulties using NSPs, found services easy to access, anonymous, discreet and they valued the advice. However, they also reported users collecting supplies on behalf of friends who were afraid of being recognised. Elsewhere 44% of IPED users obtained needles on behalf of others and 27% acquired needles from friends [54]. In one study of 1716 internet forum posts, it was evident, although not explicitly stated, that NSPs and anti-aging clinics were being used since experienced IPED users advised inexperienced users to access such services [44].

Table 5 shows that some IPED users did access HiV tests and/or vaccinations for BBVs; although take up was not high. Those who had discussed their AAS use with a doctor were more likely to have undertaken a test for Hep B or C, or HiV [8] and one study found that people screened for Hep B or C and HiV were more likely than those who did not to rate their overall experience with the doctor as good [56]. AAS users also sought advice on safer injecting.

### Research and information seeking

As Table 6 highlights IPED users’ general information about IPED use was sought from a range of sources particularly internet sites and subject specific fora. Only four studies evidenced AAS users seeking information from medical professionals [36, 49, 52, 57]. Rowe et al. [8] found that NSP staff were perceived as the most reliable source of information relating to IPED followed by nurses and doctors, however others found doctors’ knowledge limited [36, 38, 58]. For more specific information around cycling and stacking (i.e. what combination of substances are used over what length of time), dealers, fellow users and online fora were utilised. Maycock and Howat [36] found that experienced users and dealers were seen as a credible source of information. This is not without risk as substances may affect individuals differently, dependent on physiological make-up and patterns of use. One study found that over 60% of AAS users reported getting incorrect information about adverse side-effects from credible sources [36] and some AAS users acknowledged that not all information from dealers was reliable [43]. Additionally, one study highlighted self-experimentation as a key method for working out the most efficacious doses [3].

**Table 6:** Data by type of information or support - Research and information seeking

Type of information / support	Support sought from (if given)	Article reference
Seeking of general information on IPED use: including effectiveness, dosage, the effects, how to use, types of substances/brand	Friend(s)/Experienced user(s)/Training partners/Peers/Other user(s)/Family	[1, 2, 4, 8, 12, 13, 15, 16]
	Online forums*	[3, 8, 17]
	Underground books/Magazines	[1, 8, 13, 16, 18]
	Doctor(s)/Medical practitioner(s)/Nurse(s)	[1, 4, 12, 15]
	Gym contact(s)/Gym trainer(s)/Personal trainers	[1, 4, 12, 15, 19]
	Dealer(s)/Supplier(s)	[1–3, 11, 12, 15]
	Questions to anti-doping hotline/Online service on AAS	[20]
	Internet/Specialist websites*	[2–4, 12, 13, 15, 16, 18]
	Medical journals	[1]
	NSP(s)	[2, 4, 12, 15]
	Steroid guides in gyms/Other sources	[12, 15, 18]
Research into cycling, stacking and types of substances	Peers/Fellow users	[2, 13]
	Websites	[16]
	Dealers	[2]
	Online forums	[17]
	Self-experimentation	[16]
Research into side effects and risk management	People with ‘hands-on’ experience of use/ Steroid guides in gyms/ Underground books/Dedicated websites	[18]
	Questions to anti-doping hotline/Online service on adverse side-effects/ Health risks	[20]
Doping tests	Questions to anti-doping hotline/Online service on obtaining positive doping test and penalties	[20]

Any data given about access of services that is not linked to AAS/IPED use has not been included in this table. *It could be that when AAS users refer to websites they might also mean specialist forums

### Support for health issues

Some studies referenced IPED users ensuring that they got their ‘bloods’ checked, and other tests done regularly by a medical professional (Table 7) however, not all had told their doctor about their IPED use [8, 19, 52, 58]. In some countries, IPED users were able to access prescription medicines [42, 59]. IPED users sought help from Accident and Emergency departments and NSPs and self-medicated for AAS-related health issues [19] but it is unclear which, if any information sources they accessed on how to self-treat. Help was sought from experienced users [38] often through online fora [41]. All six AAS users in Skårberg et al.’s study [38] were using an addiction clinic to help manage their AAS use/dependency specifically to support psychological problems. Differences were found in the type of support or information sought dependent on the type of participant and type of support offered. Women were more likely to access health services than men, and older men were more likely to access these than younger men [55].

**Table 7:** Data by type of information or support – Support for health issues

Type of information/support	Support sought from (if given)	Article reference
Regular medical check-ups / Unspecified laboratory/Medical tests including blood-tests	Not stated Not stated but bloodwork obtained	[21] [22]
	Doctor(s) Doctors (Liver function test, ECG, Diabetes tests)	[4] [12] [9] [14]
	Steroid Clinic (service provider information)	[11]
	Anti-aging clinics	[9]
Consultation on specific AAS – related health issues	Doctor Doctor (includes discussion on mood) Doctor (for PCT advice)	[21] [6] [5] [9] [14] [23]
	Specialised addiction clinic (psychological problems)	[13]
	Sexual health clinics	[5]
	NSPs NSPs (including < 1% interventions – drug treatment referrals)	[6] [5] [7]
	Accident & Emergency/walk-in	[5] [6]
	Anti-aging clinics	[9]
	Self-treatment and other	[6]
	Online websites/Forums	[9]
Prescribed substances relating to AAS use	Not stated	[6]

Any data given about access of services that is not linked to AAS/IPED use has not been included in this table

### Ideal support

**Fig. 2:**
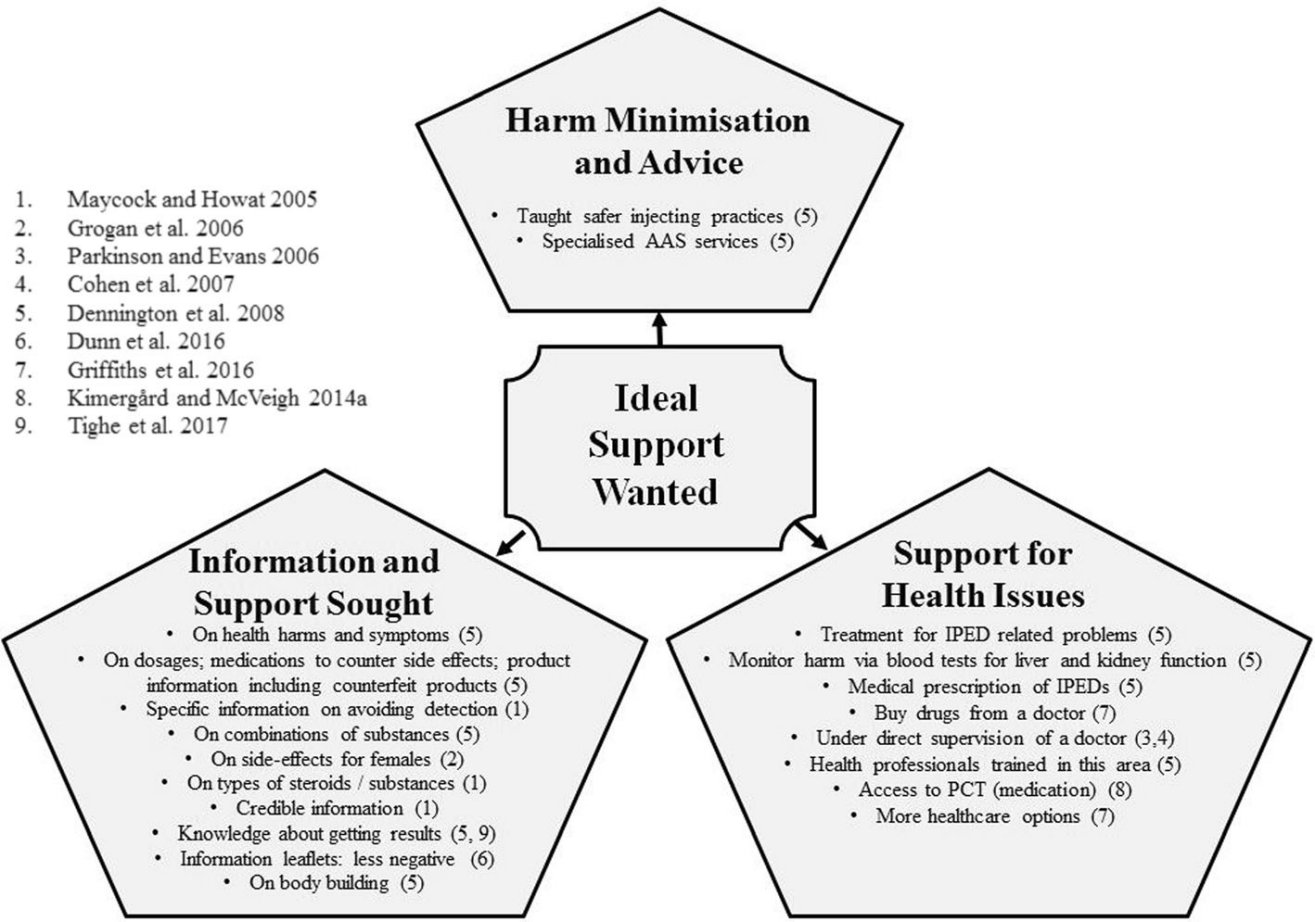
Ideal Support Wanted

Figure 2 lists the kind of support that IPED users wanted.

One study found that people who were thinking about using AAS sought out detailed information to make informed choices [36]. Requests were posted on internet fora for information on side-effects and the most effective ways to achieve results [44]. According to Dennington et al. [33] users wanted to know the optimum way to use IPED, where to acquire high quality substances, effective nutrition and exercise regimes, safer injecting techniques, safe ways to combine substances for effectiveness and when to consult a doctor. Grogan et al. [37] reported that women found much of the online information and ‘steroid bibles’ male-centric and wanted more information on side-effects for females.

In one study 66% of participants were willing to seek medical supervision for their AAS use [58], and 91.6% of users wished to use AAS legally under direct supervision of a knowledgeable doctor [5]. Some AAS users were frustrated by the limited health options available and were willing to buy drugs from their doctor [41]. Users expressed a desire for treatment for IPED-related physical problems, e.g. abscesses and the need for specific services such as blood screening [33, 39]. Griffiths et al. [42] found that AAS users wanted post-cycle therapy (PCT) to stay healthy, minimise harms and to prevent losing the gains acquired from use. Furthermore, a few users suggested that IPED should be legal and medically prescribed [33]. Some users wanted specialist IPED services where drugs could be tested for purity and to know how to avoid counterfeit drugs [33, 36]. The ideal support sought was focussed on managing health risks [5, 41]. Moreover, participants were also specific about how that support should be delivered, wanting: 1. a place to obtain credible advice and information that was non-judgemental and balanced and 2. medical support by knowledgeable professionals.

## Discussion

In summary, it is clear a large number of AAS users seek out information and support, predominantly from online fora and from experienced AAS users. Professionals are trying to tailor support to AAS users where resources allow but few studies have explicitly asked users what type of support they need. There is potentially a large number of AAS users who have not been surveyed as they are not accessing local substance use services or choose not to complete surveys for fear of being classified as ‘junkies’ [60].

One key purpose of a review is to identify gaps in the literature [61] and IPED users seemed to reject the ‘medical model’ that doctors are the experts as they give credibility to advice from people who have used [33] stating that doctors lacked credibility as they did not have personal experience [36]. This perspective is more aligned to a social care perspective with the substance user being the expert in their own use, hence the trust in experienced users. One reason given for this lack of credibility was that IPED users felt that the advice from professionals was not balanced and focused on health harms whilst ignoring the benefits [33]. Many argue for professionals to be better informed [53, 55] so as to be able to challenge the doses in ‘steroid bibles’ [37]. In a society where men are affected by images of the idealised male body image [62–64], and negative messages from others, it is unsurprising that men adopt a range of strategies to become more muscular [65–67]. Many of the short-term effects of AAS use are reversible and not as life-threatening as the long-term effects and the severity of side-effects could be reduced with early access to health services [55]. Consequently, having the appropriate support in place for AAS users is vital and some recommend that peers could have a positive role in harm minimisation [68].

The literature was sparse on the support that women access and want; this was not unexpected as the majority of AAS users are male [69]. Dennington et al’s. report [33] was the only one to include transgender people. This is a population that has not traditionally been identified within the research, but one small study found that transgender youth had 26.6 times greater odds of AAS use without a prescription than cisgender male respondents [70]. It is worth considering that this group may be using AAS as part of the transition from female to male [71], but this is not necessarily the case and therefore more research on support for women and the transgendering population would be useful, particularly aligned to support needs.

### Online information

Many users sought AAS information from the internet, but the majority of online material presents a pro-use position [72], can be incorrect or even dangerous [73] and sites may sell steroids [74], which could put users at risk and could perpetuate the impetus to use. Andreasson and Johansson [75] suggest that the online community with its openness and acceptance of AAS use is part of a culture of learning and education for novices. They believe such communities can be seen to normalise AAS use, the idea of obtaining an ‘ideal masculine body’ without using AAS becoming a fantasy.

### Support services

Most support from professionals has a harm minimisation focus. AAS users are already less likely than traditional injecting substance users to engage in risky injection practices [76] which could explain the low uptake for BBV tests. However, AAS use does increase sex drive [77] so this could increase sexual risk taking and may explain why HIV tests uptake was higher than BBVs. Users also sought help from sexual health clinics [53]. If, however, IPED users do not perceive this as a risk, they may not be engaging with services, and might be accessing NSPs simply because the needles are free. Three studies evidenced that guidance on injecting came from AAS dealers [8, 40, 43]. This is concerning as dealers often trivialised potential risks [37]. A good harm minimisation strategy could be for gyms to provide a safer injecting service [78] and this outreach service has been provided in some UK gyms [39]. However, gyms are often reluctant to provide anything that would suggest that their clientele may be using AAS [79]. For people who wish to access PCT there are few services available. Hence the need to reconsider PCT support due to the perceived needs linked to mental and physical health [42].

Only two studies [38, 55] showed that AAS users seek support for potential mood changes or underlying psychological issues. Kanayama et al. [69] concluded on the basis of seven studies that 30% of illicit AAS users develop dependence based on the Diagnostic and Statistical Manual of Mental Disorders (DSM) IV criteria and therefore it is a valid diagnostic entity. The DSM 5 [80] states that some individuals with muscle dysmorphia (MD), a form of body image disturbance, use AAS. Moreover, one study found that men using AAS for image-related reasons reported higher levels of MD and eating disorder symptomology [79, 81] suggesting there is a need for more awareness raising and that people showing such symptoms should be supported through appropriate gender specific interventions [82]. No study evidenced a need for support aligned to stopping AAS use. Traditionally, UK substance misuse support services offer talking treatments, and group and one-to-one sessions for people dependent on substances, yet there was no evidence in the UK studies of AAS users accessing these services.

Previous studies have advocated that specialist steroid services, created with input from AAS users are needed [39]. There are comparatively few specialised support services for people who use AAS and those few dedicated Steroid Clinics, often publicly-funded harm reduction initiatives, are subject to the ‘whims’ of local funding and resourcing. It would be useful to investigate ways of engaging AAS users with health services [6]. A useful strategy could be through health professionals engaging with online fora as a mechanism for harm reduction providing the language used is that of the forum and not of health professionals [44]. This would need to include strategies to overcome the lack of trust AAS users have in professionals. This review echoes these recommendations and suggests that there is a case to consider AAS users as a different population to traditional substance users. The AAS users accessing NSPs are more likely to be those who are injecting AAS and not those who take AAS orally. People who only use oral AAS are therefore potentially an even harder to reach population who are nevertheless putting themselves at risk. Dennington et al.’s [33] report examining current users’ views on the information and support provision found opposing views on types of support offered depending on the individual perspective of the user. Recent studies have identified distinct types of AAS user, each with different motivations for use [59, 83]. Differing motivations could be one reason why AAS users have differing opinions on the support offered. Consequently, offering information and support through a range of services and mediums and targeted at the different types of AAS use could be beneficial.

### Barriers to accessing support

This review did not explore why people may not access the information and support that is currently available to them. However, several studies highlighted reasons as to why AAS users chose not to access specific services. When it came to accessing NSPs, pharmacies, and doctors, AAS users spoke of a fear of stigma or embarrassment [33, 39, 41, 43, 55], and there were several other reasons given for not accessing professional services [33, 36, 37, 41, 42, 55, 58]:

- perceived lack of trust or lack of knowledge from professionals
- fear of judgemental reactions
- inability to obtain drugs wanted for PCT
- the need for private health insurance
- cost and difficulty of booking advance appointments
- not wanting to be identified as ‘drug’ users or as visiting such support services

Generally, AAS users do not see themselves as “typical” drug users [33, 43]. Consequently, a key barrier for accessing NSPs [33] was the presence of other types of substance users. Another consideration could be the link between AAS use and MD [84, 85] as research suggests that people with MD may be in denial of this as a problem [86] and may not link it to their use of AAS. A lack of recognition of an underlying psychological problem would mean AAS users would not naturally seek any type of psychological support.

Using AAS requires more preparation, research and planning than other illicit drug use, and users take a strategic approach looking to minimise harm and maximise results [58]. This could explain why AAS users justify their use as being different from other types of people who use illicit substances. Whilst many felt a stigma in attending NSPs, others felt these offered a discreet service [41]. This area of barriers to accessing services requires further investigation.

### Weaknesses and strengths

As the search was limited to English language papers, this could have excluded some studies. In studies where participants were recruited from NSPs, the authors have presumed that AAS users were accessing those services, predominantly to obtain injecting equipment. Another limitation is that data came from different countries, which influences information and support available and willingness to take part in surveys, e.g. AAS use in Australia and America is illegal, whereas in the UK, it is legal for personal use, but it is illegal to supply. A further challenge has been to identify the types of substances used within the literature and exactly what information and support is related to which substance. However, as it is likely that people who use AAS are also using these in combination with a number of other substances to either achieve their aims or mitigate side effects, it is plausible that the support and information they seek is similar. To our knowledge, this is the first scoping review on the types of support accessed, and support wanted.

## Conclusion

AAS users access a wide range of sources to obtain information on: injecting, effectiveness of substances, dosages to use, side effects, cycling and stacking, and risk management, which suggests that this is the type of information users want. AAS users seek out support from medical professionals and NSPs for health issues, blood tests, prescription substances, and equipment, suggesting these types of support are wanted by AAS users. However, AAS users do not state or potentially recognise a need for psychological support, or support to stop using. Consideration of the barriers faced by users for accessing services identified a need for services to take a non-judgemental approach and have credible knowledge around use. There is a need for AAS support to be specific and targeted, with further research required to understand their experiences around drug-use and their support needs. More research into the experiences of female and transgender AAS users and the stigma all AAS users experience would be beneficial to ensure a less ‘one size fits all’ service provision. Providers of services need to have an in-depth knowledge of benefits, harms and range of drugs available and benefits of PCT. This review echoes previous studies regarding the need to gain a deeper understanding of methods that would encourage AAS users to seek support.

## Additional file

Additional file 1: Database Search. (DOCX 16 kb)

## Data Availability

Data sharing is not applicable to this article as no datasets were generated or analysed during the current study.

## Abbreviations

AAS: Anabolic Androgenic Steroids
BBVs: (Blood Borne Viruses)
IPED: Image and Performance Enhancing Drugs
MD: Muscle Dysmorphia
NSP: Needle and Syringe Programmes
PCT: Post Cycle Therapy
