# Supplementary material for: Support for people who use Anabolic Androgenic Steroids: A Systematic Scoping Review into what they want and what they access
Source: BMC Public Health. 2019 Jul 31;19:1024. doi: 10.1186/s12889-019-7288-x (PMC6670144; doi:10.1186/s12889-019-7288-x)
Supplement: Supplementary file 1 — Database Search. (DOCX 16 kb) [file 12889_2019_7288_MOESM1_ESM.docx]

**Appendix 1 – Database Search**

**-** EBSCO Search of 114 databases and Scopus

54 of the databases searched (without any exclusion inclusion criteria) returned data– plus separate Scopus search: June 2018. **Total: 6384**

| **All Providers** | **No.** | **All Providers** | **No.** |
| --- | --- | --- | --- |
| InfoTrac Newsstand | 1,191 | HeinOnline | 12 |
| Academic Search Complete | 901 | Library, Information Science & Technology Abstracts | 10 |
| MEDLINE Complete | 699 | eBook Collection (EBSCOhost) | 9 |
| Complementary Index | 627 | eBook Academic Collection (EBSCOhost) | 9 |
| SPORTDiscus with Full Text | 508 | PsycARTICLES | 7 |
| Supplemental Index | 430 | Communication Source | 6 |
| PsycINFO | 354 | British Library EThOS | 6 |
| CINAHL Complete | 284 | J-STAGE | 5 |
| ScienceDirect | 224 | Cochrane Database of Systematic Reviews | 5 |
| SocINDEX with Full Text | 215 | JSTOR Journals | 5 |
| Regional Business News | 211 | Credo Reference: Academic Core | 5 |
| Education Source | 113 | Hospitality & Tourism Complete | 4 |
| Business Source Complete | 110 | GreenFILE | 3 |
| Directory of Open Access Journals | 56 | Art & Architecture Complete | 3 |
| Newswires | 51 | Government Publishing Office Catalog | 3 |
| NewsBank | 41 | Communication Abstracts | 2 |
| Networked Digital Library of Theses & Dissertations | 34 | Bournemouth University Library Catalogue | 2 |
| LexisNexis Academic: Law Reviews | 32 | Informit Literature & Culture Collection | 2 |
| ERIC | 31 | IEEE Xplore Digital Library | 2 |
| Environment Complete | 28 | PsycBOOKS | 1 |
| SciELO | 25 | arXiv | 1 |
| Harvard Library Bibliographic Dataset | 24 | Informit Health Collection | 1 |
| Teacher Reference Center | 21 | Hoover's Company Profiles | 1 |
| SwePub | 18 | Alexander Street Press | 1 |
| British Library Document Supply Centre Inside Serials & Conference Proceedings | 18 | Digital Access to Scholarship at Harvard (DASH) | 1 |
| OpenDissertations | 18 |  |  |
| NewsBank - Archives | 12 | Scopus (searched separately) | 1687 |
